# Flexible Perovskite Solar Cells via Surface-Confined Silver Nanoparticles on Transparent Polyimide Substrates

**DOI:** 10.3390/polym11030427

**Published:** 2019-03-06

**Authors:** Xiangfu Liu, Lin Hu, Rongwen Wang, Junli Li, Honggang Gu, Shiyuan Liu, Yinhua Zhou, Guoli Tu

**Affiliations:** 1 Wuhan National Laboratory for Optoelectronics, Huazhong University of Science and Technology, 1037 Luoyu Road, Wuhan 430074, China; xfliu@hust.edu.cn (X.L.); hulin@hust.edu.cn (L.H.); wrw@hust.edu.cn (R.W.); lijunli@hust.edu.cn (J.L.); yh_zhou@hust.edu.cn (Y.Z.); 2 State Key Laboratory of Digital Manufacturing Equipment and Technology, Huazhong University of Science and Technology, Wuhan 430074, China; hongganggu@hust.edu.cn (H.G.); shyliu@hust.edu.cn (S.L.)

**Keywords:** transparent polyimide, perovskite solar cells, surface-confined silver nanoparticles

## Abstract

We report about a flexible substrate incorporating surface-confined silver nanoparticles on transparent polyimide (PI). The incorporated silver nanoparticles (Ag NPs), which possessed excellent adhesive strength with the PI substrate, induced localized surface plasmon resonance and light scattering effects by changing the particle size and interparticle distance to promote light harvesting in the perovskite solar cells. Moreover, the reduced sheet resistance was beneficial for the charge extraction and transportation in the devices when high-conductivity poly(3,4-ethylenedioxythiophene):poly(styrene sulfonate) (PEDOT:PSS, PH1000) was deposited on the Ag NP-confined PI serving as a flexible bottom electrode. A power conversion efficiency of 10.41% was obtained for the flexible perovskite solar cells based on a Ag NP-confined PI substrate (the particle size of the Ag NPs was 25 nm mixed with 40 nm), which was obviously enhanced in all parameters. Especially, a 61% improvement existed in the short-circuit current density compared to that based on the bare PI substrates. It indicates that the substrate would be a promising candidate for the development of flexible electronics.

## 1. Introduction

Emerging technology products require high-standard flexible and transparent substrates [1,2,3,4,5]. During the past few decades, much effort has been devoted to the development of plastic substrates, including polycarbonate [6,7,8,9], poly(ethylene terephthalate) [10,11,12], poly(ethylene naphthalate) [13,14,15], polyester sulfone [16,17], and transparent polyimide (PI) [18,19,20]. Among these substrates, transparent PIs have attracted immense interest as next-generation intelligent electronic equipment because of their excellent thermal stability and a high glass transition temperature (Tg) of over 300 °C, as well as superior chemical/mechanical properties [21]. Perovskite solar cells (PSCs) with excellent efficiency in excess of 22% are emerging as a promising candidate for future photovoltaic technology [22,23]. The reported current density of PSCs is still below the theoretical value of approximately 26 mA cm^−2^ [24], which indicates that a large amount of incident sunlight is not utilized in the process of photocurrent generation. To solve this problem, numerous light-trapping schemes using periodic or random plasmonic structures, such as metallic nanoparticles (MNPs), have been proposed [25,26,27,28,29,30,31,32]. The localized surface plasmon resonance (LSPR) of an MNP is determined by the particle material, size, shape, and interparticle distance, which can induce light harvesting/scattering and consequent near-field enhancement of electric fields [33,34,35,36,37,38,39]. Currently, the reported MNPs in solar cells are normally utilized by being incorporated into the solution of the interfacial or absorber layer. These protocols always cause undesirable dispersion and weak adhesion of the MNPs, which complicate roll-to-roll preparation and mass production [40,41,42,43,44]. 

In this report, a novel, flexible substrate was developed by incorporating surface-confined silver nanoparticles (Ag NPs) on transparent polyimide (PI) via all-wet chemical processes (hereafter referred to as “Ag NP-confined PI substrate”). The Ag NPs were chemically adhered onto the surface of the PI film to form a robust structure. We optimized the Ag NPs with a size of 25 nm mixed with 40 nm to achieve favorable localized surface plasmon resonance and light scattering effects for the flexible substrates to promote light harvesting in the perovskite solar cells. Moreover, the sheet resistance of the high-conductivity poly(3,4-ethylenedioxythiophene):poly(styrene sulfonate) (PEDOT:PSS, PH1000) electrode was notably reduced when deposited on the Ag NP-confined PI substrate than on the bare PI substrate, which would facilitate the charge extraction and transportation in the devices. A power conversion efficiency (PCE) of 10.41% was thus achieved for the flexible perovskite solar cells based on the Ag NP-confined PI substrate (the particle size of the Ag NPs was 25 nm mixed with 40 nm), which is obviously enhanced compared to that based on the bare PI substrates. Especially, there was a 61% improvement in the short-circuit current density. This indicates that this substrate would be a promising candidate for the development of flexible electronics.

## 2. Materials and Methods 

### 2.1. Materials and Characterization

Potassium hydroxide (KOH), silver nitrate (AgNO_3_), dimethylamine borane (DMAB), gamma-butyrolactone (GBL), and dimethyl sulfoxide (DMSO) were purchased from Sigma-Aldrich and used as received. PEI and PC_61_BM were purchased from Xi’an Polymer Light Technology Corp (Xi’an, China). PEDOT:PSS (Clevios PH1000) and PEDOT:PSS (Heraeus CLEVIOSTM Al4083) were purchased from Hereaus (Beijing, China). CH_3_NH_3_I and PbI_2_ for the perovskite were obtained from Materwin New Materials Technology Co., Inc (Beijing, China). Polyimides in the form of polyamic acid were provided by Nanjing IMIDE Optoelectronic Materials Technology Co., Ltd (Wuhan, China).

The Fourier transform infrared (FTIR) spectra were measured using an FT/IR 670 (VERTEX 70, Bruker, Karlsruhe, Germany) equipped with an attachment for attenuated total reflection (ATR). The surface morphology of the samples was characterized by standard scanning electron microscopy (SEM) analysis (FEI Helios Nanolab 600, Columbus, Bad Oldesloe, Germany) after sputter-coating with approximately 4 nm gold. Atomic force microscopy (AFM) imaging was performed using a Veeco Dimension 3100 NanoScope in a tapping mode (Bruker RTESPA-300, Karlsruhe, Germany). X-ray diffraction (XRD) patterns were also acquired to investigate the formation and structure of the metallic Ag NPs (X’Pert Materials Research Diffractometer, Philips, Amsterdam, Netherlands). The transmittance and photoluminescence (PL) spectra were acquired using a UV-vis-NIR (UV-3600, Shimadzu, Kyoto, Japan) spectrophotometer and an Edinburgh FLS920 fluorescence spectrometer (Edinburgh Instruments, Edinburgh, UK), respectively. Time-resolved PL decay was measured using time-correlated single-photo counting instrumentation (Edinburgh FLS920, Edinburgh Instruments, Edinburgh, UK) with a 375 nm laser source.

### 2.2. Synthesis of Surface-Confined Ag Nanoparticles

First, the transparent PI films were prepared as described in a previous work [45], and carefully cleaned using an ethanol solution, followed by immersion in a 5 M aqueous KOH solution for 5 min to hydrolyze the film surface. Subsequently, the surface-cleaved PI film was immersed in a 100 mM aqueous AgNO_3_ solution at room temperature for 30 min to generate silver ions via an ion-exchange reaction. Finally, a chemical reduction of the silver ions was performed by immersion of the silver-ion-doped PI film in DMAB aqueous solution for 30 min. After each step, the film was rinsed with copious amounts of distilled water. Three types of Ag NPs adhered to the films when treated with KOH for 1, 1, and 2 h, and then reduced using 0.1, 10, and 0.1 mM DMAB: Ag (25 nm), Ag (25/40 nm), and Ag (40 nm), respectively.

### 2.3. Fabrication of a Flexible Device

A highly conductive PEDOT:PSS (PH1000) with 5 wt.% ethylene glycol and 0.1 wt.% nonionic surfactant PEG-TmDD was spin-coated on an Ag-confined PI substrate at a speed of 1000 rpm for 60 s and subsequently annealed at 140 °C for 10 min in air [46]. The hole-transporting PEDOT:PSS (4083) layer was spin-coated at 4000 rpm for 1 min, and then annealed at 120 °C for 20 min. The perovskite absorber precursor solution (1.3 M CH_3_NH_3_I and 1.3 M PbI_2_ in anhydrous GBL and DMSO with a volume ratio of 7:3) was then spin-coated onto the substrate at 4000 rpm for 30 s, following which the antisolvent toluene was immediately added at the center of the rotating surface at the 27th second. After annealing at 100 °C for 10 min, the films were coated with PC_61_BM chlorobenzene solution (20 mg mL^−1^) at 2000 rpm for 60 s. A PEI isopropanol solution (0.1 wt.%) was then spin-coated on top at 5000 rpm for 60 s. Subsequently, the samples were placed inside a vacuum evaporation system, and a silver electrode (100 nm) was evaporated with a metal aperture mask. The device area was dependent on the intersection of the bottom patterned PH1000 electrode and the top Ag electrodes. All the effective areas were corrected with an aperture and the device area was 0.04 cm^2^. The fabricated cells were measured under simulated AM 1.5G (100 mW cm^−2^) irradiation in a nitrogen atmosphere glove box. The external quantum efficiency (EQE) spectra were acquired using a 150 W xenon lamp (Oriel) equipped with a monochromator (Cornerstone 74004, Newport, San Francisco, Oregon, USA).

### 2.4. Theoretical Simulation 

The field intensity distributions of the PSCs were simulated via the finite difference time domain (FDTD) method using commercial software (FDTD solution, Lumerical Inc., Vancouver, Canada). For all the devices, polarized incident light propagating in the z-direction with polarized incident light at wavelengths of 460, 600, and 780 nm, using perfectly matched boundary layers in all dimensions except for the top Ag electrode with metal boundary conditions, was determined. The scattering intensity was monitored by a total field power monitor and a scattered field power monitor. It was assumed that the Ag NPs were perfect spheres and were in point contact with the polyimide substrate. Moreover, the distance between the particles was set from 10 to 20 nm and evaluated based on SEM results. The size of the NPs was varied from 25 to 40 nm, based on the empirically derived average size and its variation. The refractive indices (n) and k values for each material were measured using a spectroscopic ellipsometer (ME-L, Wuhan Eoptics Technology Co., Ltd, Wuhan, China) in the reflection measurement mode with the incident angle set at 65°. The film thickness of all the layers was set based on the experimentally determined values using a stylus profilometer (Dectak XT, Bruker, Karlsruhe, Germany) and cross-section SEM. 

## 3. Results and Discussion

**Figure 1:**
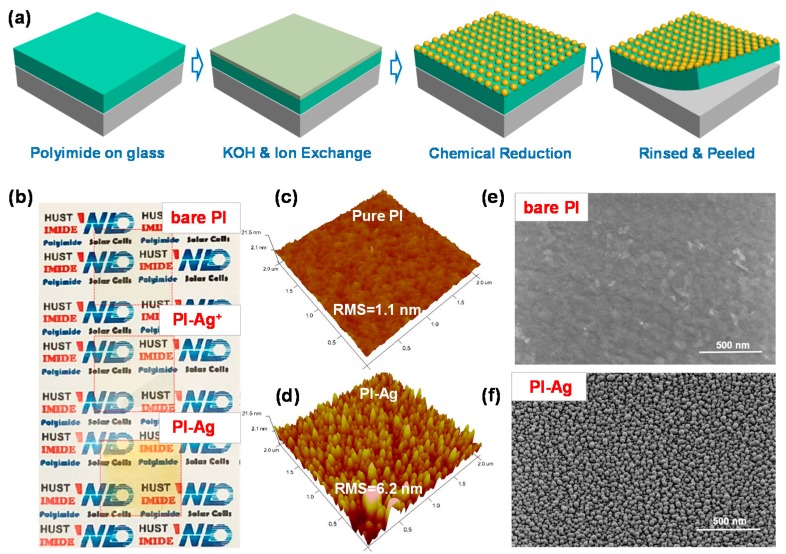
(**a**) The fabrication process of the surface-confined silver nanoparticle (Ag NP) polyimide (PI) substrate. (**b**) Photograph of the bare polyimide film, and films before and after dimethylamine borane (DMAB)-induced reduction of the ion-doped precursors. (**c**,**d**) The atomic force microscopy (AFM) image (2 × 2 μm) of bare and DMAB-reduced PI. (**e**,**f**) Scanning electron microscopy (SEM) image of the bare and DMAB-reduced PI.

The preparation process for the Ag NP-confined PI substrate via an all-wet preparation is described in detail in the Materials and Methods. A schematic is shown in Figure 1a. The PI films on glass were hydrolyzed with aqueous potassium hydroxide (KOH) to generate a thin layer of potassium salts of carboxylic acid and amide bonds on the surface of the PI [47,48]. Then, the silver ions were introduced onto the surface-modified layer by immersing the substrates in silver nitrate (AgNO_3_) aqueous solution to induce an ion exchange with the potassium ions. Finally, the precursor films loaded with Ag ions were treated with aqueous dimethylamine borane (DMAB) solution for chemical reduction. The Ag NP-confined PI substrate was then rinsed with abundant deionized water for use. There was no obvious difference in the appearance of the silver ion-doped precursor film and the original PI film. After immersion in the DMAB solution for 20 min, the colorless film gradually changed to a canary yellow silver color (Figure 1b), indicating the Ag ions reduced to form Ag NPs on the surface of the film. 

Figure S1 further shows the ATR-FTIR spectra of the bare PI and surface-modified films that were treated with KOH, AgNO_3_, and DMAB aqueous solutions. The symmetric and antisymmetric carbonyl stretching of the imide rings with characteristic bands at 1780 and 1710 cm^−1^ disappeared completely after ring cleavage by KOH treatment. New bands appeared at 1500–1700 cm^−1^, which can be attributed to the complexation of K ions with the carboxyl groups (1500-1600 cm^−1^), as well as the amide bond involved in the carbonyl stretching (1650 cm^−1^) and N–H bending (1530 cm^−1^). After ion exchange and reduction treatment, although the spectrum was similar to that of the KOH-treated film, a mild decrease in the intensity at 1650 cm^−1^ implies that a number of corresponding states substituted for the Ag^−^ and Ag complexes. AFM and SEM were performed to observe the surface morphology change during Ag modification. As shown in Figure 1c,e, the bare PI substrate had a root-mean-square (RMS) roughness of 1.1 nm and an extremely smooth surface. After treatment with KOH and AgNO_3_, many shallow grooves emerged (Figures S2 and S3). The RMS of the PI films increased to 6.2 nm (Figure 1d), and essentially exhibited full coverage of Ag NPs (Figure 1f) after DMAB reduction. As a result, we found that lengthening the treatment time (from 0.5 to 8 h) with the KOH and increasing the concentration of DMAB (from 0.001 to 10 mM) increased the Ag NP size (range 10 to 60 nm, Figure S4) and interparticle distance (±5 to ±30 nm, Figure S5). 

**Figure 2:**
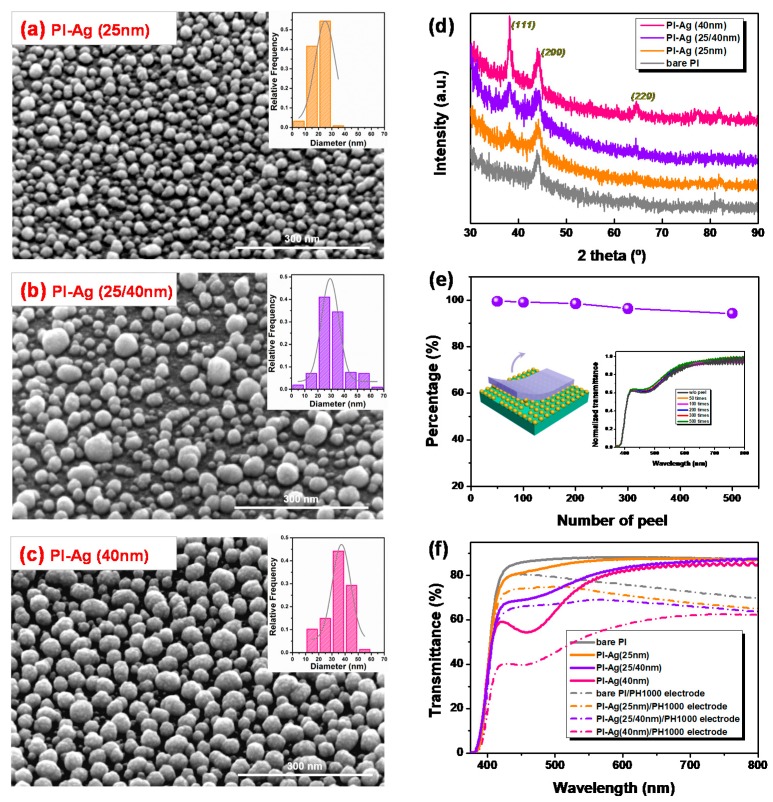
(**a**–**c**) The scanning electron microscopy (SEM) image of PI with the various sizes of Ag nanoparticles (inset: the size distribution histogram showing the diameter of ~25, ~25/40 and ~40 nm, respectively). (**d**) X-ray diffraction (XRD) patterns for PI film with different sizes of Ag nanoparticles. (**e**) The adhesive strength of the Ag covered on the surface-modified PI (inset: the transmittance spectrum of the Ag-modified PI films with various numbers of peels). (**f**) Optical transmittance of the bare PI and PI adhered with different sizes of Ag nanoparticles (solid line), and their corresponding transmittance after coating the PH1000 electrodes (dash line).

To explore whether the size and distance between the Ag NPs adhered to the surface of the PI influenced the performance of PSCs, SEM imaging was performed and the results are presented in Figure 2a–c. Three kinds of Ag particles with diameters of ~25 nm, ~40 nm, and 25 nm mixed with 40 nm, with interparticle distances of 15, 10, and 20 nm, respectively, were identified on the PI surface (see details in Materials and Methods). XRD patterns were collected to confirm the size of the NPs based on the emergence of the (111) peaks reflecting at 38°, due to the face-centered cubic (FCC) structure of the Ag NPs [49]. The lower diffraction pattern in Figure 2d indicates that there were no (111) peaks for the bare PI substrate. The increased peak intensity and reduced full width at half maximum corresponded to an increase in the size of the Ag particles, which agrees with the SEM result.

**Figure 3:**
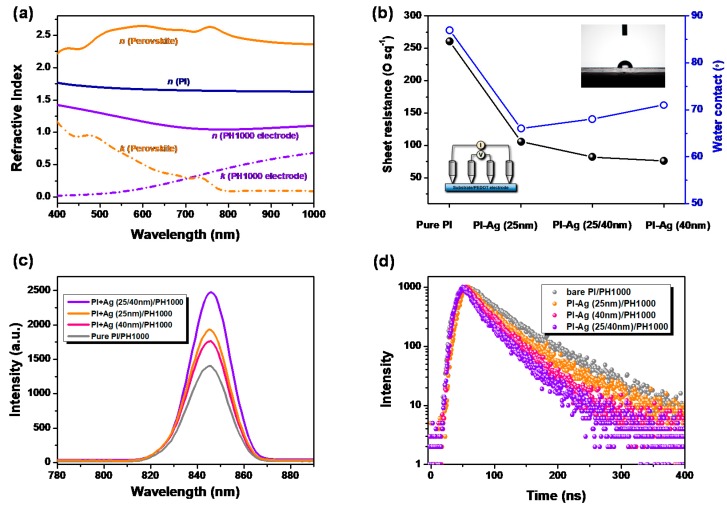
(**a**) The refractive index (n) and extinction coefficient (k) of the perovskite layer, PH1000 electrode, and PI substrate. (**b**) The surface hydrophilicity of the bare PI and Ag-modified PI substrate, and the corresponding sheet resistance (*R_sh_*) after coating the PH1000 electrode. The inset shows the water contact measurement and four-point probe system used to measure the surface property and *R_sh_*. (**c**) Photoluminescence of perovskite film based on bare PI and Ag-modified PI substrate coating with PH1000 electrode, and (**d**) the corresponding time-resolved PL spectrum.

Peel-off experiments were employed to investigate the adhesive strength of the Ag NPs that had formed on the surface of the PI. A sticky tape was pasted firmly onto a PI-Ag surface and then peeled by hand. As shown in Figure 2e, the plasmon resonance absorption of the Ag NPs remained at 98.5% after 200 peels and 94.3% after 500 peels with sticky tape. When the PI-Ag films were tested by ultrasonic treatment in water, the residual quantities of Ag nanoparticles were 96.1% after 0.5 h and 85.9% after 1 h, as shown in Figure S6. These results indicate that the Ag NPs possessed excellent adhesive strength with the PI substrate, which could be beneficial in upper device layer processing during device fabrication. Figure 2f illustrates the optical transmittance of the PI substrates with the Ag-modified layers. Due to the plasmonic resonance absorption of the Ag NPs, the transmittance of the film at 460 nm decreased as the size of the Ag NPs increased (from 87% in bare PI to 81% with 25 nm, 70% with 25/40 nm, and 55% with 40 nm). Subsequently, the coated PH1000 applied as the electrode herein [50] shifted its corresponding values to 80%, 73%, 66%, and 40%, respectively. It is observed that the plasmon resonance absorption in the Ag NPs weakened the optical transmission of the PI-Ag substrates to a certain extent. Nevertheless, compared with the Ag NPs with a low refractive index (nAg ≈ 0.14 at 460 nm), the PI, PH1000, and perovskite layer exhibited a high refractive index (nPI ≈ 1.73, nPH1000 ≈ 1.36, and nperovskite ≈ 2.29 at 460 nm, Figure 3a), which can change the direction and phase of the incident light at their interfaces and induce scattering and interference, consequently resulting in a shift in the optical distribution in the perovskite absorber layer. Moreover, the incorporation of the Ag NPs could reduce the sheet resistance (*R_sh_*) of the PH1000 electrodes from 261 Ω/sq for bare PI to 106, 82, and 76 Ω/sq in PI-Ag substrates along with the increasing particle size, respectively, as plotted in Figure 3b. This may be attributed to the improved wettability of the Ag-confined surface, resulting in preferential compatibility with the upper PH1000 electrode (Figure S7). Additionally, the coated PH1000 could connect the isolated islands of the Ag NPs and enhance their conductivity. Thus, *R_sh_* of the PH1000 electrode on PI-Ag (40 nm) was lower than those of PI-Ag (25/40 nm) and PI-Ag (20 nm).

The room temperature-steady PL spectra for perovskite film on bare and Ag-confined PI substrates are presented in Figure 3c. The PL intensity of the Ag-modified samples was enhanced with respect to the reference sample when excited at 460 nm. The enhanced PL intensity was ascribed to the excitation of LSPR, which enhanced the degree of light absorption, and the light excitation rate is the occurrence of strong interactions between the plasmonic field and excitons [51,52,53]. A faster decay of the lifetime of the perovskite films based on these corresponding substrates was observed in the time-resolved PL measurement (Figure 3d). The obvious change after the incorporation of the Ag NPs was on account of the strong coupling between the excitons and the plasmonic field as previously reported. The plasmon–exciton coupling participated in charge transfer, thus facilitating exciton dissociation into free polarons [54]. Thereby, the existence of the Ag NPs on PI is beneficial for both exciton dissociation and recombination reduction in the perovskite absorber.

**Figure 4:**
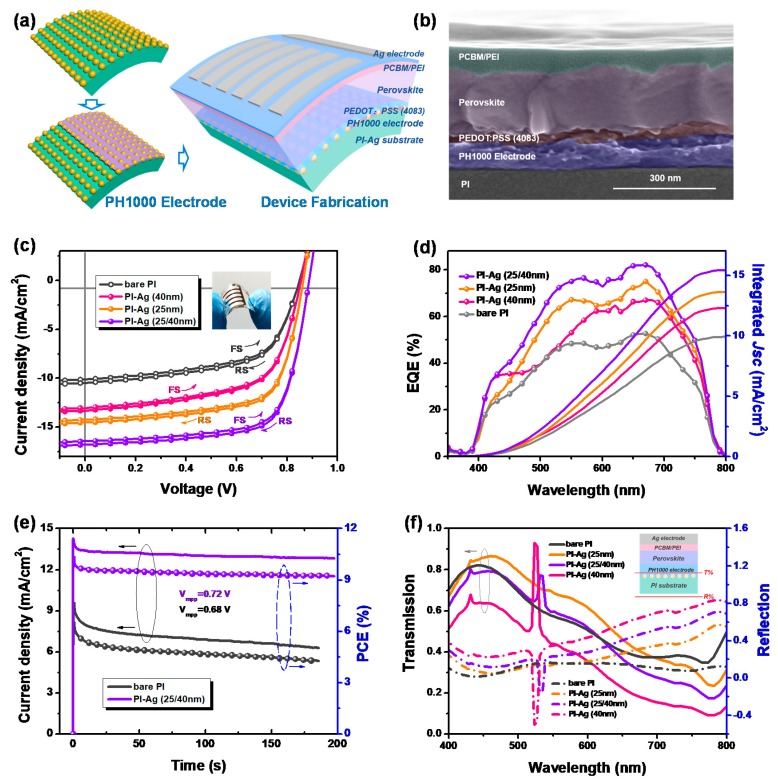
(**a**) Schematic of the fabrication of a flexible PH1000 electrode and perovskite solar cells; (**b**) SEM cross-sectional image of perovskite solar cells; (**c**) J–V curves of flexible perovskite solar cells with a structure of bare PI or PI-Ag substrates/PH1000 electrode/PEDOT:PSS (4083)/CH_3_NH_3_PbI_3_/PC_61_BM/PEI/Ag measured in both the reverse and forward directions (inset: the picture of bending PSCs); (**d**) their corresponding EQE spectra and integrated current density; (**e**) stabilized power output of perovskite solar cells with bare PI and PI-Ag (25/40nm) substrates; (**f**) optical analysis of the reflection and transmission in the devices (inset: the monitor position of the cells).

In order to evaluate the Ag NP-confined PI substrate, perovskite solar cells with a structure of PI-Ag/PH1000 electrode/PEDOT:PSS (4083)/CH_3_NH_3_PbI_3_/PC_61_BM/PEI/Ag were fabricated (Figure 4a,b). The control device with glass/ITO as the substrate displayed typical performance, as previously reported (Figure S8 and Table S1) [55]. For comparison, a flexible device based on the bare PI substrate was also used, which displayed a PCE of 5.41%, short-circuit current density (*J_sc_*) of 10.56 mA cm^−2^, open-circuit voltage (*V_oc_*) of 0.84 V, and fill factor (FF) of 61%, as shown in Figure 4c and Table 1. There was a significant enhancement in the PCE, reaching 6.63% for PI-Ag (40 nm) and 8.13% for the PI-Ag (25 nm) substrate after introduction of the Ag NPs. The optimal flexible device prepared on the PI-Ag (25/40 nm) substrate exhibited a 60.9% increase in *J_sc_*—up to 16.80 mA cm^−2^—a PCE of 10.41%, *V_oc_* of 0.88 V, and FF of 70%. These enhancements for the flexible PSCs indicate that the coverage of the Ag NPs contributed significantly toward improving the electrical and optical properties as mentioned above, thereby directly promoting the photocurrent and device efficiency. The EQE spectra of the PSCs with and without the Ag NPs were measured and are presented in Figure 4d. It is evident that the trend in the EQE enhancement of the cells was in accordance with that of the *J-V* curves. In particular, the adhesion of Ag (25/40 nm) to the PI substrate acquired an obvious enhancement in the EQE spectra compared with that of the bare PI device in the wavelength range of 420–780 nm. Although the Ag (40 nm)-confined PI showed the optimal *R_sh_*, the lowest transmittance caused by plasmon absorption at approximately 460 nm hindered the achievement of a better overall performance. The integrated *J_sc_* values from the EQE spectra were also consistent with that of the J-V measurement (Figure 4d). It is noted that a stable power output of approximately 10% was achieved for the PI-Ag device after applying a bias at the maximum output voltage, as shown in Figure 4e. That can be ascribed to the surface-confined Ag NPs that build the robust connections between the PI substrate and upper organic layer. We also stored the flexible devices in an air-filled dryer without encapsulation to compare their stability. As shown in Figure S9, the device based on PI-Ag substrates only delivered a 50% PCE after 10 days and the device based on bare Ag was even worse. The decayed device performance cannot be attributed to only the perovskite itself but also the contact interface between the PH1000 electrode and the flexible substrates. A phenomenon that the flexible PI substrate tends to wrinkle after the subsequent annealing processes would cause a series of porosities at the interface. Moisture and oxygen, therefore, could easily permeate into the device to decay the perovskite film. A thin layer of Ag nanoparticles on the PI seemed to make the results better.

**Figure 5:**
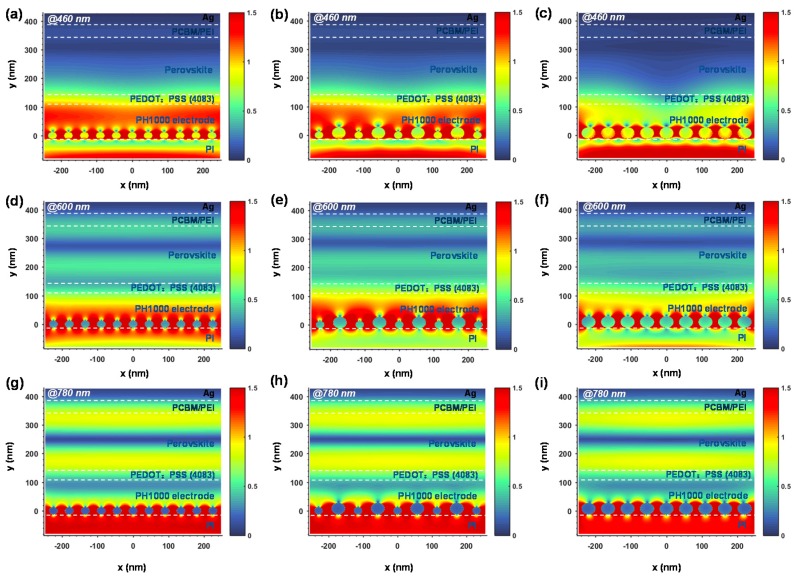
Simulated cross-section near-field distributions for TE-polarized incident light at 460 nm (top row), 600 nm (middle row), and 780 nm (bottom row) in PSCs on PI substrates with (**a**–**c**) Ag (~25 nm), (**d**–**f**) Ag (~25/40 nm), and (**g**–**i**) Ag (~25/40 nm).

To further clarify the inside optical effects of the size of the Ag NPs on light manipulation in the PSCs, theoretical calculations of light propagation were performed via the FDTD method (the detailed procedure is presented in the Materials and Methods). The optical intensity in air and the PH1000 electrode is demonstrated in Figure 4f. As a result, among the Ag NP-confined PI devices, the light reflection into air increased and the optical transmission into the upper PH1000 electrode layer decreased along with an increasing Ag NP size. In contrast to the bare PI, the PI-Ag (25 nm) substrate obtained a better transmission from 400 to 700 nm on account of the particle light scattering. The PI-Ag (40 nm) showed a worse result due to the strong light reflection and plasmonic absorption. Notably, an optical transmission peak appeared around 530 nm, which could be ascribed to the plasmon resonance [56]. Integrated with Ag (25 nm) and Ag (40 nm), the PI-Ag (25/40 nm) substrate acquired an approximately equal transmission with the bare PI from 400 to 650 nm and also possessed plasmon resonance around 530 nm. We should note that the resonant features here were different from that seen in the simulated transmission spectra from Figure 2f. The reason can be attributed to the position of the simulated monitor and the UV monitor in our experiment, which were different, while the plasmon resonance caused by the nanoparticles only localized in dozens of nanometers. The normalized electrical field distributions in the PSCs for incident light at wavelengths of 460, 600, and 780 nm are plotted in Figure 5. The PI substrate with the Ag NPs could obviously shift the near-field distributions of the incident light inside the PSCs and change the field distributions in the perovskite absorbers in contrast to that in the bare PI substrate (Figure S10). The simulation results clearly illustrate that the enhanced electrical field distribution region of the PI-Ag (25/40 nm) and PI-Ag (40 nm) due to the LSPR effect were larger than PI-Ag (25 nm). Compared with the lower plasmon effect for the 25 nm Ag NPs (Figure 5a,d,g), the optical field was shifted toward to the perovskite absorbers in the PI-Ag (25/40 nm) (Figure 5b,e,h) at approximately 600 and 780 nm. And the stronger light reflection and plasmon absorption impelled the PI-Ag (40 nm) substrate possessing a weaker field intensity (Figure 5c,f,i), which is in agreement with the EQE result in Figure 4d. These results indicate that Ag NPs could confer strong light scattering capability and plasmon resonance, which facilitate more light to be confined in the perovskite absorber. Meanwhile, much work remains on optimizing the balance between the optical transmittance and the plasmon resonance with the incorporation of Ag NPs.

## 4. Conclusions

In summary, a facile all-wet process for surface-confined Ag NPs on a transparent PI film as a flexible substrate was applied to PSCs. The experimental and theoretical results confirmed that the coverage of Ag NPs, which possessed an excellent adhesive strength with PI, was more favorable than the bare PI substrate for light harvesting enhancement in the perovskite absorber due to the SPR effect and particle scattering. The Ag-confined PI could also reduce the sheet resistance of the flexible PH1000 electrode and facilitate charge carrier extraction from the perovskite layer, mainly due to the wettability improvement and LSPR effect. Relative to the reference cell with the bare PI substrate, the incorporation of Ag NPs with a diameter of 25/40 nm and the largest interparticle distance of 20 nm resulted in an enhancement of 60% in the short-circuit current. Moreover, the PCE of the CH_3_NH_3_PbI_3_-based PSCs improved from 5.41% to 10.41%. The fabrication of an Ag-adhered PI substrate widens its potential application in flexible PSCs, with the objective of realizing wearable electronics.

## Figures and Tables

**Table 1:** Device parameters of perovskite devices based on different substrates.

Substrate	Scan direction	*J_sc_* (mA/cm^2^)	*V_oc_* (V)	FF	PCE (%)
bare PI/PH1000	RS	10.44 ± 0.39 (10.56)	0.82 ± 0.03 (0.84)	0.60 ± 0.04 (0.61)	5.13 ± 0.79 (5.41)
	FS	10.09 ± 0.40 (10.21)	0.82 ± 0.03 (0.84)	0.60 ± 0.04 (0.61)	4.96 ± 0.81 (5.23)
PI-Ag (40nm)/PH1000	RS	13.09 ± 0.35 (13.42)	0.83 ± 0.03 (0.85)	0.61 ± 0.03 (0.62)	6.63 ± 0.78 (7.07)
	FS	12.97 ± 0.37 (13.15)	0.83 ± 0.03 (0.85)	0.61 ± 0.03 (0.62)	6.57 ± 0.80 (6.93)
PI-Ag (25nm)/PH1000	RS	14.37 ± 0.35 (14.65)	0.85 ± 0.03 (0.86)	0.67 ± 0.03 (0.68)	8.18 ± 0.76 (8.56)
	FS	14.09 ± 0.36 (14.36)	0.85 ± 0.03 (0.86)	0.67 ± 0.03 (0.68)	8.02 ± 0.78 (8.39)
PI-Ag (25/40nm)/PH1000	RS	16.80 ± 0.28 (16.90)	0.87 ± 0.03 (0.88)	0.69 ± 0.03 (0.70)	10.08 ± 0.75 (10.41)
	FS	16.34 ± 0.33 (16.54)	0.87 ± 0.03 (0.88)	0.69 ± 0.03 (0.70)	9.80 ± 0.74 (10.18)

The averages for photovoltaic parameters of each device are given in parentheses with the mean variation obtained from 10 devices; the ± refers to the standard deviation. The values in parentheses state the optimal values.

## Supplementary Materials

The following are available online at https://www.mdpi.com/2073-4360/11/3/427/s1. Figure S1. ATR-FTIR spectrum of bare polyimide before and after KOH treatment, and subsequent ion exchange and DMAB chemical reduction; Figure S2. SEM images of bare polyimide before and after KOH treatment, and subsequent ion exchange and DMAB chemical reduction; Figure S3. AFM images (2 × 2 μm) of bare polyimide before and after KOH treatment, and subsequent ion exchange and DMAB chemical reduction; Figure S4. SEM images of the polyimide films subjected to 10 M KOH treatment at 50 °C for 0.5, 1, 2, 4, and 8 h and subsequent Ag ion exchange and reduction in 0.1 mM DMAB at room temperature; Histogram of Ag nanoparticle size distributions; Figure S5. SEM images of the polyimide films subjected to 10 M KOH treatment at 50 °C 1 h and subsequent Ag ion exchange and reduction in 0.001, 0.01, 0.1, 1, and 10 mM DMAB at room temperature; Histogram of Ag nanoparticle size distributions; Figure S6. The Ag surface adhesion test on the PI by (inset: the transmittance spectrum of the Ag-modified PI after ultrasonic treatment with various times); Figure S7. The contact angle of water on the bare PI and PI adhered with various sizes of Ag nanoparticles; Figure S8. (a) J–V curves of reference perovskite solar cells with glass/ITO/PEDOT:PSS (4083)/CH3NH3PbI3/PC61BM/PEI/Ag measured in both the reverse and forward directions. (b) The corresponding EQE spectra of the device and its integrated current density; Figure S9. The stability of the optimal flexible devices stored in an air-filled dryer without any encapsulation; Figure S10. Simulated cross-section near-field distributions for TE-polarized incident light at (a) 460 nm, (b) 600 nm, and (c) 780 nm in PSCs on bare PI substrates, Table S1. Device parameters of perovskite devices with glass/ITO substrate.
