# Supplementary material for: Flexible Perovskite Solar Cells via Surface-Confined Silver Nanoparticles on Transparent Polyimide Substrates
Source: Polymers (Basel). 2019 Mar 6;11(3):427. doi: 10.3390/polym11030427 (PMC6473467; doi:10.3390/polym11030427)
Supplement: Supplementary file 1 [file polymers-11-00427-s001.pdf]

Supplementary Materials

Flexible Perovskite Solar Cells via Surface-Confined Silver Nanoparticles on Transparent Polyimide Substrates

Xiangfu Liu [1,†], Lin Hu [1,†], Rongwen Wang [1], Junli Li [1], Honggang Gu [2], Shiyuan Liu [2], Yinhua Zhou [1] and Guoli Tu [1,*]

[1] Wuhan National Laboratory for Optoelectronics, Huazhong University of Science and Technology, 1037 Luoyu Road, Wuhan 430074, China; xfliu@hust.edu.cn (X.L.); hulin@hust.edu.cn (L.H.); wrw@hust.edu.cn (R.W.); lijunli@hust.edu.cn (J.L.); yh_zhou@hust.edu.cn (Y.Z.)

[2] State Key Laboratory of Digital Manufacturing Equipment and Technology, Huazhong University of Science and Technology, Wuhan 430074, China; hongganggu@hust.edu.cn (H.G.); shyliu@hust.edu.cn (S.L.)

† Xiangfu Liu and Lin Hu contributed equally to this work.

* Correspondence: tgl@hust.edu.cn

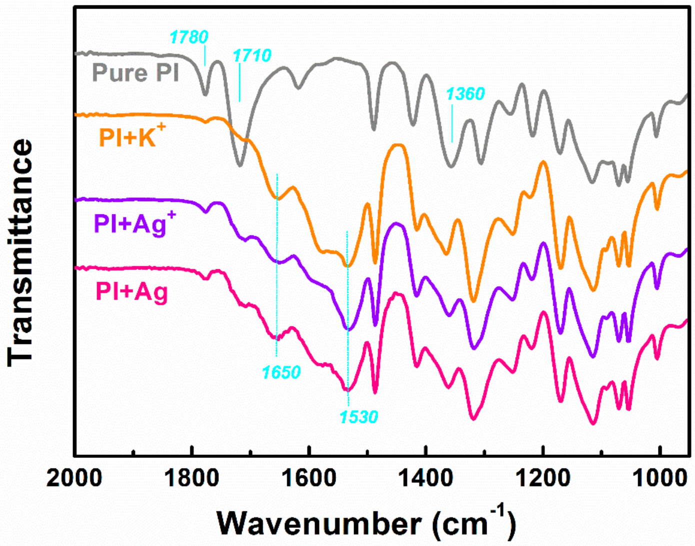

Figure S1. ATR-FTIR spectrum of bare polyimide before and after KOH treatment, subsequent ion exchange and followed by DMAB chemical reduction.

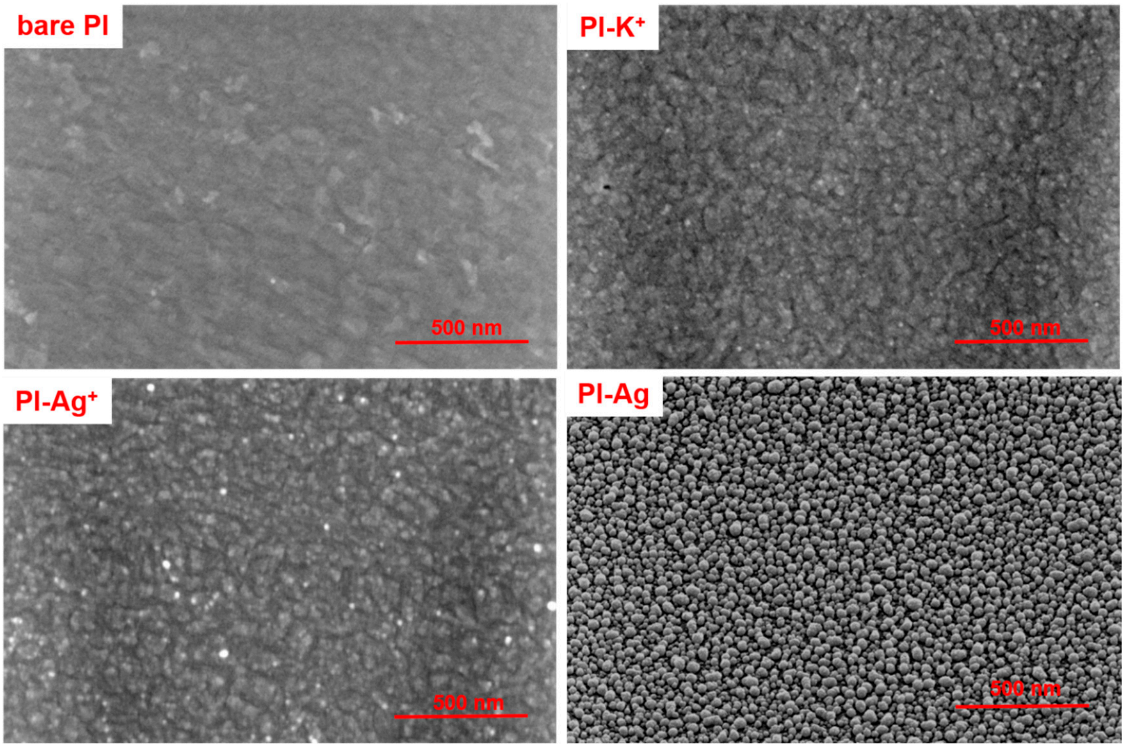

Figure S2. SEM images of bare polyimide before and after KOH treatment, subsequent ion exchange and followed by DMAB chemical reduction.

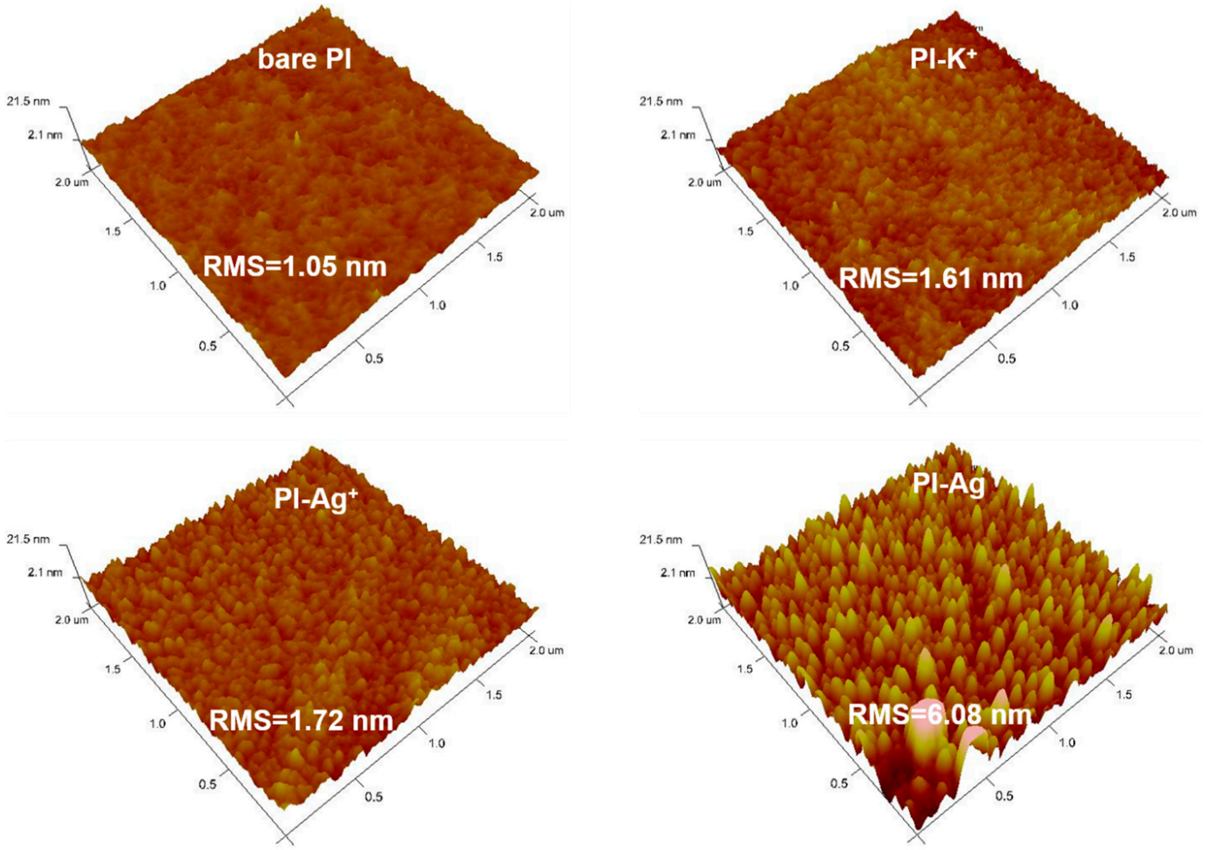

Figure S3. AFM images (2×2 μm) of bare polyimide before and after KOH treatment, subsequent ion exchange and followed by DMAB chemical reduction.

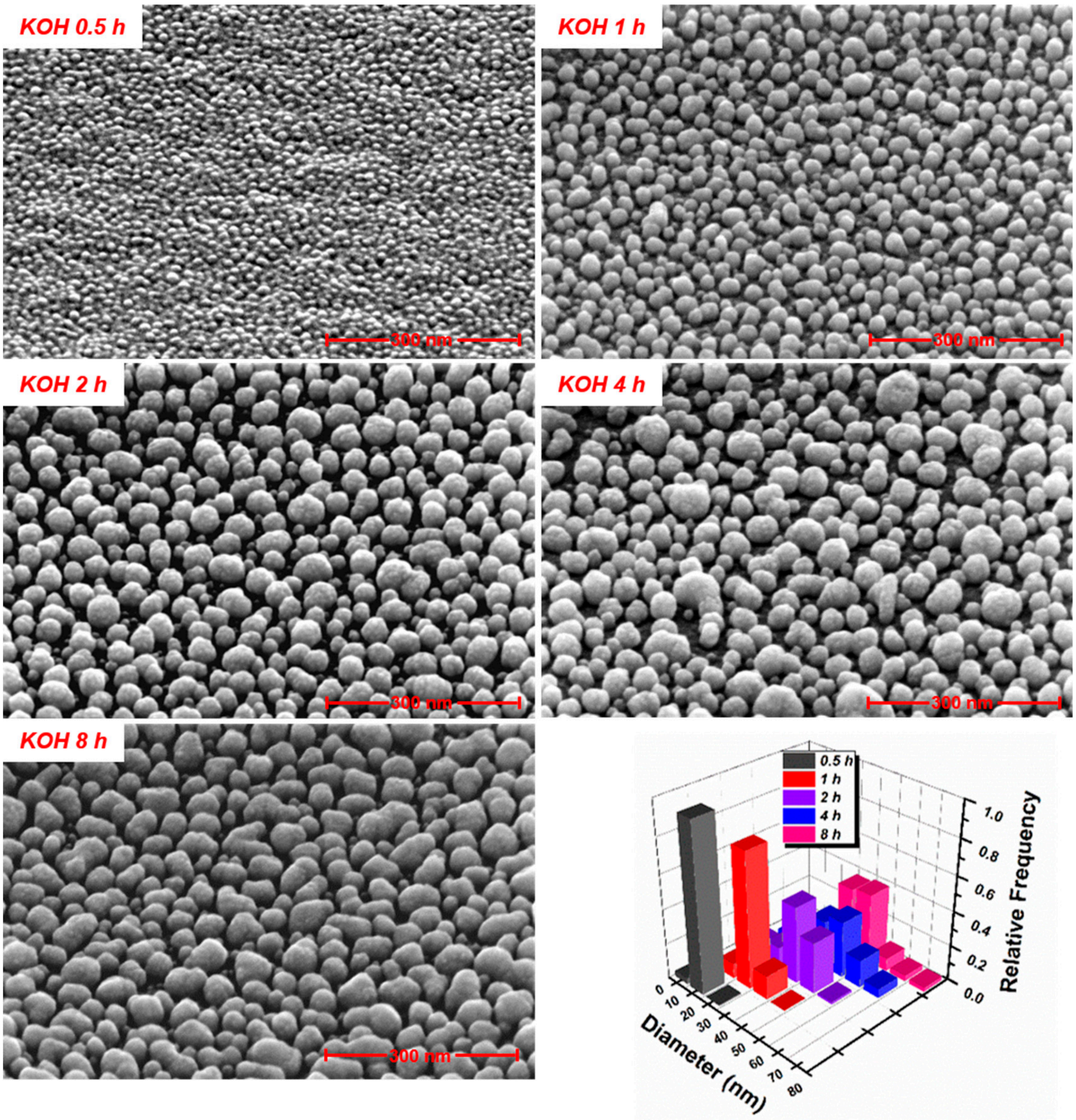

Figure S4. SEM images of the polyimide films subjected to 10 M KOH treatment at 50 °C for 0.5, 1, 2, 4, and 8 h and subsequent Ag ion exchange and reduction in 0.1 mM DMAB at room temperature; Histogram of Ag nanoparticle-size distributions.

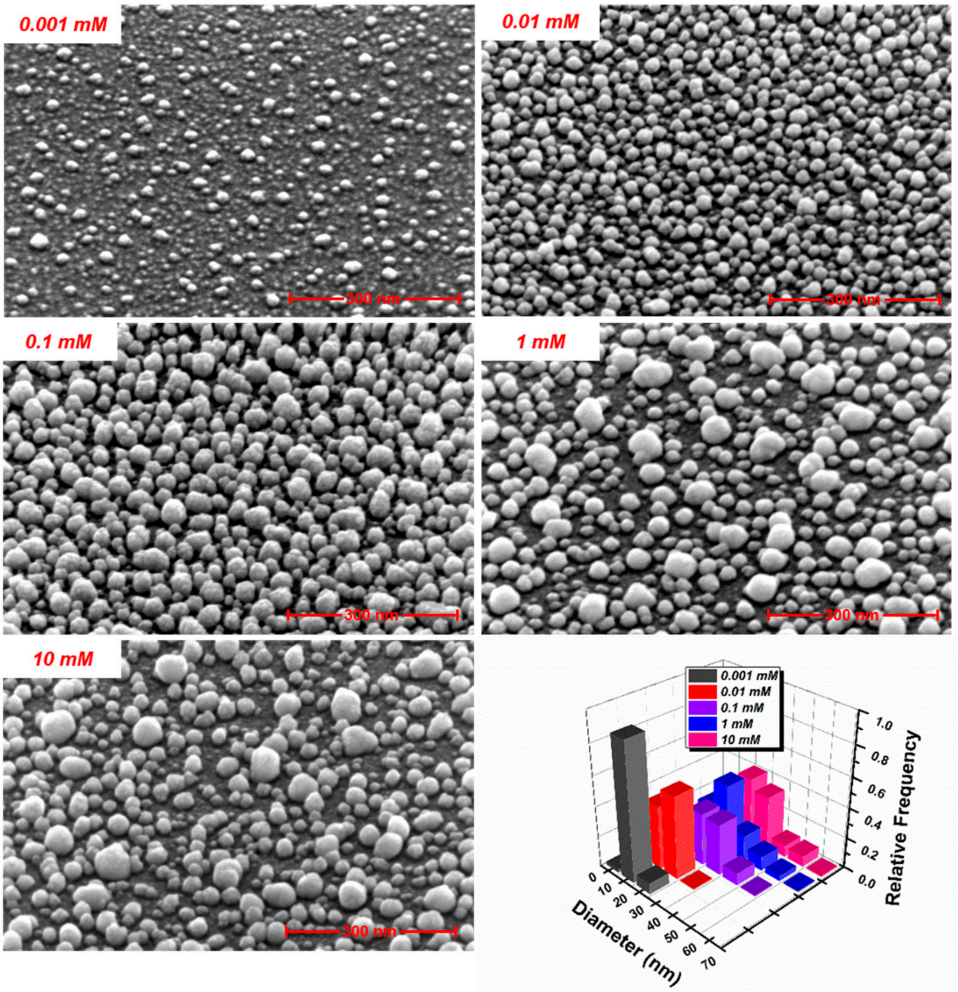

Figure S5. SEM images of the polyimide films subjected to 10 M KOH treatment at 50 °C 1 h and subsequent Ag ion exchange and reduction in 0.001, 0.01, 0.1, 1, and 10 mM DMAB at room temperature; Histogram of Ag nanoparticle-size distributions.

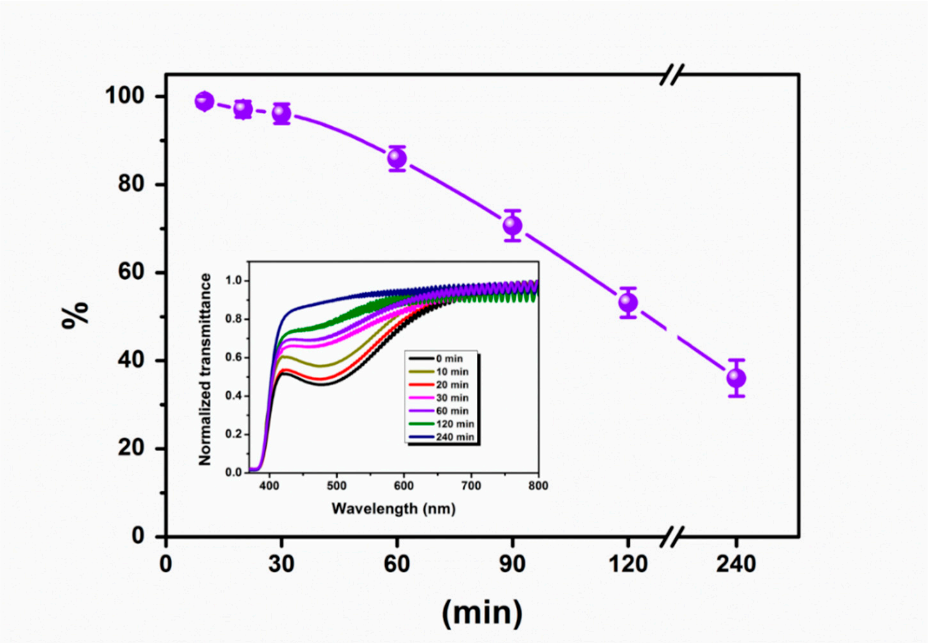

Figure S6. The Ag surface adhesion test on the PI by (inset: the transmittance spectrum of the Ag-modified PI after ultrasonic treatment with various times).

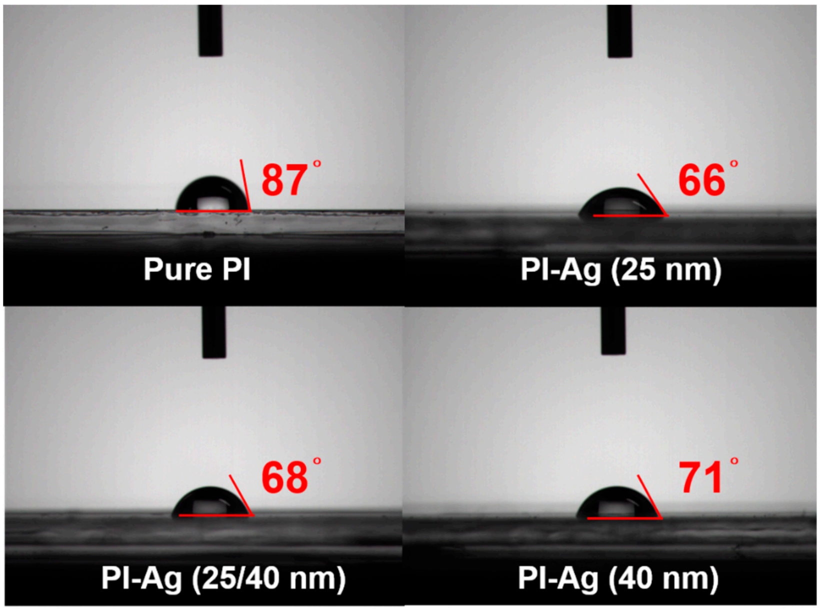

Figure S7. The contact angle of water on the bare PI and PI adhered with various size of Ag nanoparticles.

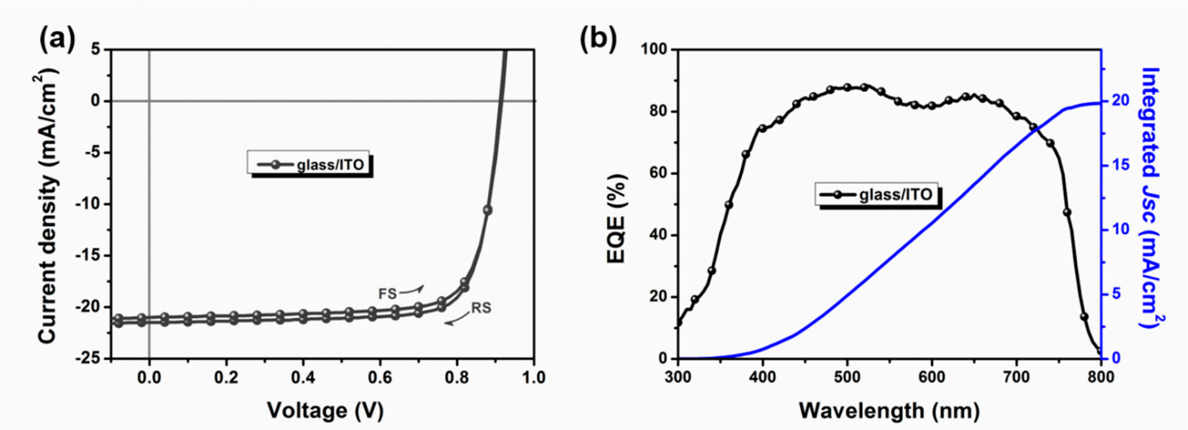

Figure S8. a) *J-V* curves of reference perovskite solar cells with glass/ITO/PEDOT:PSS (4083)/$CH_3NH_3PbI_3$/$PC_{61}BM$/PEI/Ag measured in both the reverse and forward directions. b) The corresponding EQE spectra of the device and its integrated current density.

Table S1. Device parameters of perovskite devices with glass/ITO substrate.

Substrate	Scan direction	J_{sc} (mA/cm^2)	V_{oc} (V)	FF	PCE (%)
Glass/ITO	RS	21.32±0.20 (21.57)	0.91±0.03 (0.91)	0.76±0.03 (0.78)	14.74±0.71 (15.31)
	FS	20.97±0.31 (21.10)	0.91±0.03 (0.91)	0.76±0.03 (0.78)	14.50±0.73 (14.97)

*The averages for photovoltaic parameters of each device are given in parentheses with mean variation obtained from 10 devices, and the ± refer to the standard deviation. The values in parentheses statethe optimal values.

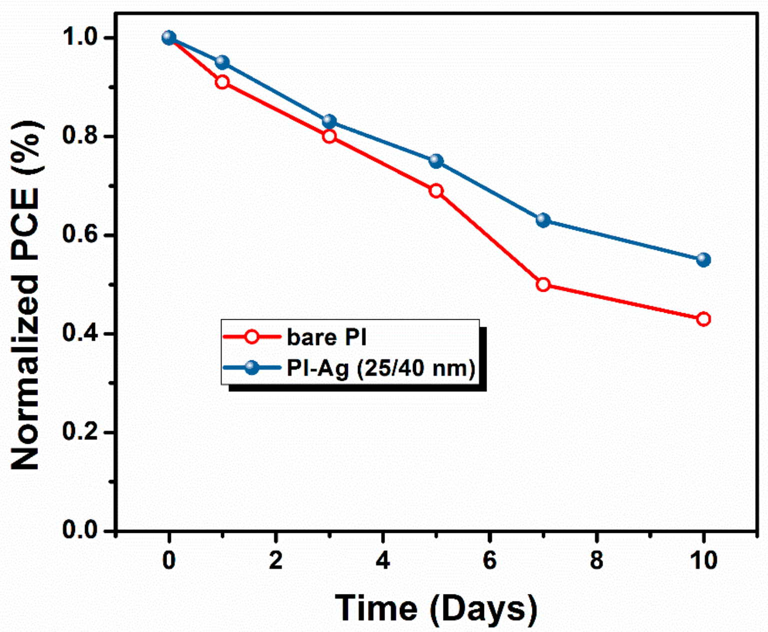

Figure S9. The stability of the optimal flexible devices stored in an air-filled dryer without any encapsulation.

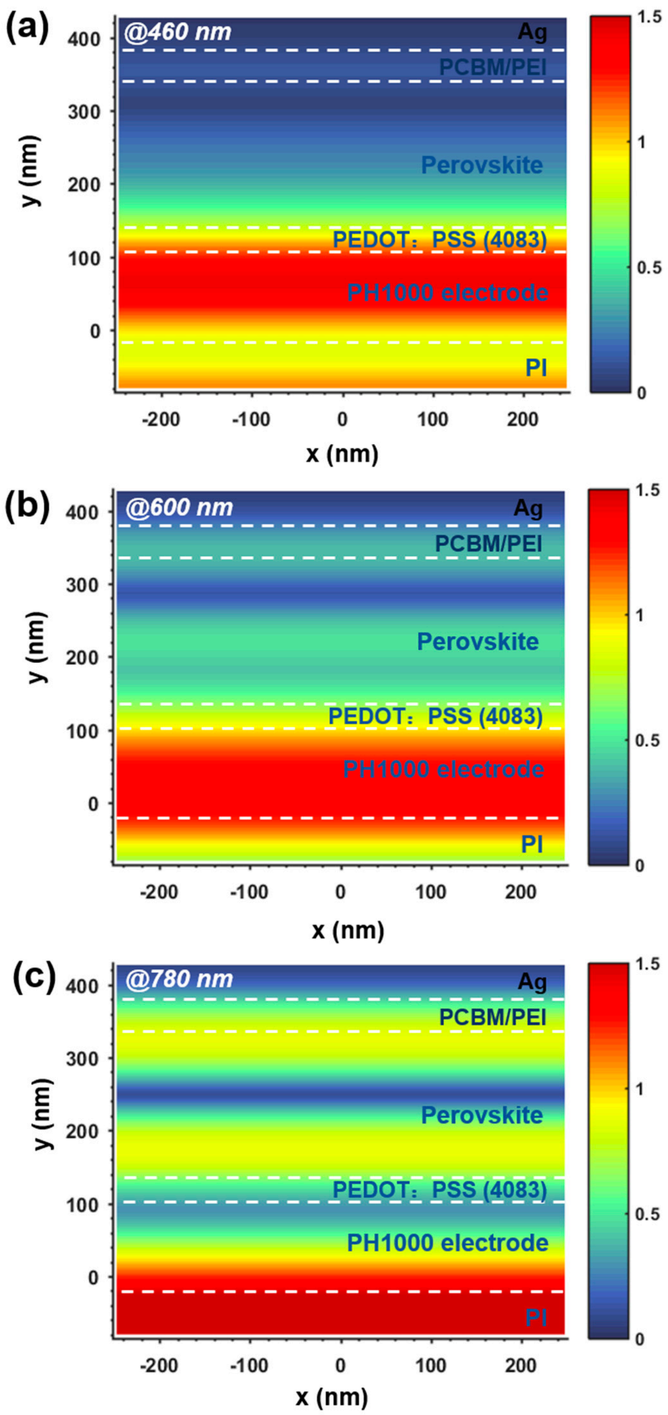

Figure S10. Simulated cross-section near-field distributions for TE polarized incident light at a) 460 nm, b) 600 nm, and c) 780 nm in PSCs on bare PI substrates.
